# A new taxonomy of neuroprotective agents for stroke appropriate for the reperfusion era

**DOI:** 10.3389/fneur.2024.1514924

**Published:** 2025-02-05

**Authors:** Mersedeh Bahr-Hosseini, Jeffrey L. Saver

**Affiliations:** Department of Neurology and Comprehensive Stroke Center, David Geffen School of Medicine at UCLA, Los Angeles, CA, United States

**Keywords:** neuroprotection, acute stroke treatment, taxonomy, cerebroprotection, clinical trial design, reperfusion era

## Abstract

The advent of the era of highly effective reperfusion therapy for acute ischemic stroke has reawakened interest in neuroprotective treatments as they are far more likely to be efficacious as synergistic complements to reperfusion rather than standalone interventions. However, testing neuroprotective agents combined with reperfusion mandates not only renewed conduct of trials but also a fundamental reconceptualization of the subclasses of neuroprotection therapies. We propose a new taxonomy of neuroprotective treatment agents appropriate for the reperfusion era that recognizes six broad classes of agents, each targeting a distinct process and time epoch of injury: (1) Bridging neuroprotectives slow infarct expansion in the pre-reperfusion period, (2) Blood–brain barrier stabilizers restore the integrity of BBB before and early after reperfusion, (3) Microcirculation lumen preservers protect arteriolar and capillary endothelial cell integrity deterring the no-reflow phenomenon, (4) Reperfusion injury preventors block inflammatory, oxidative, and other processes that start immediately after reperfusion, (5) Edema reducers avert cerebral swelling and secondary injury due to brain tissue compression and herniation, and (6) Delayed neuroprotectives mitigate injury due to apoptosis and mitochondrial dysfunction in the late post-reperfusion period. This approach also broadly distinguishes neuroprotection from other major treatment strategies, including recanalization, collateral enhancement, and neurorepair. By focusing on broad physiologic targets of action rather than granular molecular mechanisms, this six-fold classification of neuroprotection can inform the design of preclinical studies and human clinical trials, including imaging biomarker endpoint selection and treatment timing. This updated taxonomy may accelerate the translation of cerebroprotective agents from bench to bedside.

## Introduction

The elusive dream of neuroprotective treatments for acute ischemic stroke has been rekindled by the advent of highly effective reperfusion therapy. As leading classes of neuroprotective agents are better suited to the treatment of temporary, rather than permanent, ischemia, neuroprotective therapy is more likely to succeed as a synergistic complement to reperfusion treatment than as a standalone intervention. However, an obstacle to further progress is the lingering influence of outmoded frameworks of neuroprotective classes that obscure the best approaches to agent development. The coming of the reperfusion era mandates not only renewed conduct of trials of promising neuroprotective agents but also a fundamental reconceptualization of the subclasses of neuroprotection therapies.

The taxonomies of the past focused almost entirely on only one general physiologic strategy of neuroprotection—slowing the progression of cerebral injury while ischemia is ongoing. In a prototypical example, agents were classified into six categories predominantly according to the molecular mechanisms by which they putatively achieved this single physiologic goal: calcium channel blockers, glutamate antagonists, nitric oxide modulators, gamma-aminobutyric acid potentiators, free radical scavengers, and anti-inflammatory agents (1). This concentration upon molecular subclasses of agents that increase hypoxia tolerance was appropriate at a time when reperfusion was uncommonly achieved and the intra-ischemic period was perforce the predominant target of neuroprotective agent development. But is now out of date. A new taxonomy of neuroprotective treatment agents is needed appropriate for the reperfusion era.

## The proposed new taxonomy

We propose an approach that recognizes six broad classes of neuroprotection agents, each targeting a distinct mechanism of injury:

1. Bridging neuroprotectives: These agents slow infarct expansion by blocking the molecular ischemic cascade that elaborates neural injury in ischemic environments. As they act in the intra-ischemic period, they need to be administered soon after (or even prior to) stroke onset and before reperfusion therapies have restored tissue blood flow.
2. Blood–brain barrier stabilizers: These agents restore blood–brain barrier integrity. By stabilizing tight junctions and the neurovascular unit, they reduce the risk of hemorrhagic transformation and restore cerebral autoregulation. If highly effective, blood brain barrier stabilizers could permit thrombolytic drugs to be given at higher than standard doses to increase lysis efficacy.
3. Microcirculation lumen preservers: These treatments avert arteriolar and capillary endothelial cell edema, pericyte contraction, and vasospasm that decrease microcirculatory lumen size, deterring the no-reflow phenomenon (2). They complement lytic and platelet disaggregating treatments that dissolve small thrombi within these vessels and constitute a form of recanalization.
4. Reperfusion injury preventers: These therapies block inflammatory, oxidative, and other processes that cause additional neuronal cell death following achievement of reperfusion. They may be given systemically but also may be given via intra-arterial infusion directly into the reperfused field at the end of an endovascular thrombectomy procedure.
5. Ionic cytotoxic and vasogenic edema reducers: These agents modulate aquaporin and other channels that regulate brain water movement and the size of intracellular and extracellular fluid volumes. They can avert secondary injury due to brain tissue compression and herniation.
6. Delayed neuroprotectives: These therapies block mechanisms of neural injury that are elaborated over several days after the initial ictus, including programmed cell death (apoptosis, necroptosis) and mitochondrial dysfunction. When early reperfusion of tissues averts initial necrotic cell death during the first hours, a substantial proportion of the initially salvaged tissue may experience such delayed injury (3).

## Discussion

**Figure 1:**
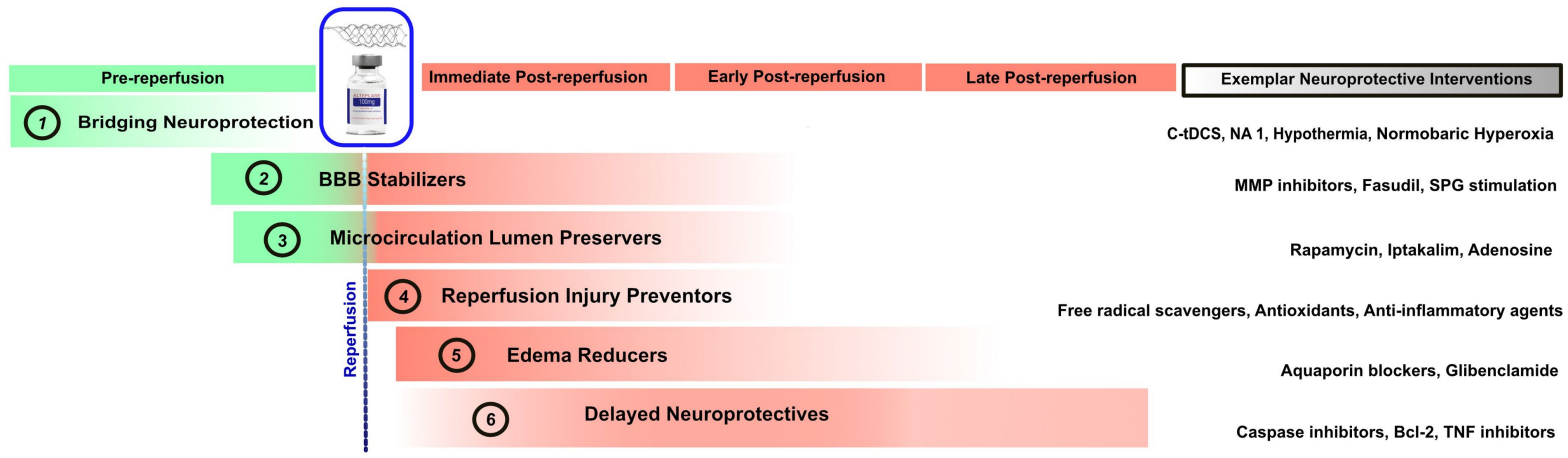
Timepoints in which the six proposed classes of neuroprotective agents exert their ameliorative effects. The rightmost column shows exemplar agents in each neuroprotective category. tDCS, Transcranial Direct Current Stimulation; NA, Nerinetide; MMP, Matrix Metalloproteinases; SPG, Sphenopalatine Ganglion; TNF, Tumor Necrosis Factor.

By focusing on broad physiologic targets of action rather than granular molecular mechanisms, this six-fold classification has direct clinical and development program relevance. Clinical trial designs that recruit a broad population of patients in different stages of ischemic injury will hamper identification of benefit when testing an agent targeted on a specific stage and mechanism of injury. Preferred timing of treatment start varies substantially across the different classes. Bridging neuroprotectives must be given hyperacutely, while ischemia is still ongoing. They therefore require trials with treatment start in the prehospital setting or soon after Emergency Department arrival. Reperfusion injury preventers are optimally started as soon as reperfusion has been achieved (although they can be started earlier so as to already be in place when blood flow is restored). Microcirculatory vessel wall protecters and blood brain barrier stabilizers should be started early, preferably even during the ischemic period but also likely can be helpful if started early after reperfusion. Edema reducers and delayed neuroprotectives can be expected to be still effective if started later, several hours after reperfusion has been established. Figure 1 depicts the proposed six neuroprotective classes, the best treatment start timing relative to reperfusion time, and exemplars of each category.

In addition, each of these broad neuroprotective classes has distinct imaging biomarkers that can provide a readout of physiologic efficacy enabling proof of concept and dose optimization in early-stage trials (4). To highlight a few among the many distinctive physiologic efficacy biomarkers available: bridging neuroprotection can be assessed by penumbral salvage; blood brain barrier stabilizers by hemorrhagic transformation rates and permeability imaging; microcirculation lumen preservers by tissue level reperfusion; reperfusion injury preventers by infarct growth despite reperfusion; ionic edema reducers by edema volume quantification; and delayed neuroprotectives by late MRI FLAIR appearance of injury after initial apparent tissue salvage on diffusion imaging. Similarly, distinctive imaging biomarkers identify patient populations who may benefit from one class of neuroprotectants. Imaging signatures of informative, target patients include: for bridging neuroprotectives, substantial penumbra on mismatch imaging; for blood brain barrier stabilizers, early permeability imaging abnormalities; for microcirculation lumen preservers, reduced tissue-level perfusion despite microcirculatory recanalization; for reperfusion injury preventers, successful reperfusion; for ionic edema reducers, large hemispheric infarction; and delayed neuroprotectives, diffusion abnormality reversal indicating severe ischemic insult but averted necrotic cell death.

While some treatments will target only a single neuroprotective process, others will pleiotropically modify more than one of these mechanisms. For example, hypothermia slows down progression to infarction, suppresses the inflammatory response, and stabilizes the blood brain barrier.

It is also important to distinguish neuroprotection from, rather than conflate it with, other acute/subacute treatment strategies for ischemic stroke, including recanalization, collateral enhancement, and neurorepair. We demur from overly broad definitional approaches that collapse two or more of these distinct approaches into a single, ungainly neuroprotection category, and obscure more than they clarify (5, 6). Recanalization therapies aid the ischemic brain by reopening macrocirculatory and microcirculatory blocked vessels to restore orthograde blood flow and end the ischemic period. Collateral enhancement therapies aid the ischemic brain by increasing blood flow through collateral channels to end, or reduce the intensity of, the ischemic period. Neuroreparative agents increase neural repair and neuroplasticity, enabling better recovery from a completed stroke though not by protecting against the original injury. These three classes of treatments yield more preserved brain or improved neural function but do so via mechanisms other than neuroprotection. Neuroprotective agents may be defined as treatments that interrupt the cellular, biochemical, and metabolic elaboration of injury during or following exposure to ischemia (7).

The rubric “neuroprotection,” as commonly employed, refers to protection of not just neurons but all elements of the brain parenchyma, including glia (glioprotection), endothelium (endothelioprotection), and the integrated neurovascular unit. The term “cerebroprotection” is formally more accurate to encompass this range of effects and has been advocated for adoption (8). However, the term “neuroprotection” remains the label predominantly used by the scientific community. (For example, in PubMed for calendar year 2023, 1,098 articles were retrieved with search terms neuroprotection and stroke compared with only 25 for cerebroprotection and stroke.)

A potential limitation of this new taxonomy is that it is designed for the use of neuroprotective agents as complementary therapy to reperfusion treatment and not for patients with permanent ischemia. Nonetheless, a substantial (15–20%) (9) and increasing proportion of acute ischemic stroke patients are now receiving reperfusion therapies in high-income countries and these patients have the most severe stroke that account for the preponderance of post-stroke disability and death (10). In addition, if the commonly held view that neuroprotection as a standalone therapy without reperfusion is a will-of-the-wisp that is not attainable, then focusing upon the concomitant reperfusion therapy setting is required (11). However, agents that possess both neuroprotective and lytic or neuroprotective and collateral enhancement remain potential standalone treatments.

In the current era of highly effective reperfusion therapy, ischemic stroke patients have much better outcomes than in the past, but still too often suffer disability or death (12). The updated taxonomy of neuroprotection here elaborated, suitable to the reperfusion era, can hasten the translation of cerebroprotective agents from bench to bedside, and further improve the care of patients with ischemic stroke.

## Data Availability

The original contributions presented in the study are included in the article/supplementary material, further inquiries can be directed to the corresponding author.

## References

[1] Davis M Barer D. Neuroprotection in acute ischaemic stroke. II: clinical potential. Vasc Med. (1999) 4:149–63. doi: 10.1177/1358836X9900400306, PMID: 10512595

[2] Sun F Zhou J Chen X Yang T Wang G Ge J. No-reflow after recanalization in ischemic stroke: from pathomechanisms to therapeutic strategies. J Cereb Blood Flow Metab. (2024) 44:857–80. doi: 10.1177/0271678X241237159, PMID: 38420850 PMC11318407

[3] Kidwell CS Saver JL Starkman S Duckwiler G Jahan R Vespa P. Late secondary ischemic injury in patients receiving intraarterial thrombolysis. Ann Neurol. (2002) 52:698–703. doi: 10.1002/ana.10380, PMID: 12447922

[4] Goyal M McTaggart R Ospel JM van der Lugt A Tymianski M Wiest R. How can imaging in acute ischemic stroke help us to understand tissue fate in the era of endovascular treatment and cerebroprotection? Neuroradiology. (2022) 64:1697–707. doi: 10.1007/s00234-022-03001-z, PMID: 35854136

[5] Haupt M Gerner ST Bahr M Doeppner TR. Neuroprotective strategies for ischemic stroke-future perspectives. Int J Mol Sci. (2023) 24:4334. doi: 10.3390/ijms24054334, PMID: 36901765 PMC10002358

[6] Mulder IA van Bavel ET de Vries HE Coutinho JM. Adjunctive cytoprotective therapies in acute ischemic stroke: a systematic review. Fluids Barriers CNS. (2021) 18:46. doi: 10.1186/s12987-021-00280-1, PMID: 34666786 PMC8524879

[7] Ovbiagele B Kidwell CS Starkman S Saver JL. Neuroprotective agents for the treatment of acute ischemic stroke. Curr Neurol Neurosci Rep. (2003) 3:9–20. doi: 10.1007/s11910-003-0031-z, PMID: 12507405

[8] Lyden PD. Cerebroprotection for acute ischemic stroke: looking ahead. Stroke. (2021) 52:3033–44. doi: 10.1161/STROKEAHA.121.032241, PMID: 34289710 PMC8384682

[9] Raychev R Sun J-L Schwamm L Smith EE Fonarow GC Messé SR. Performance of thrombectomy-capable, comprehensive, and primary stroke centers in reperfusion therapies for acute ischemic stroke: report from the get with the guidelines–stroke registry. Circulation. (2023) 148:2019–28. doi: 10.1161/CIRCULATIONAHA.123.066114 37855118

[10] Malhotra K Gornbein J Saver JL. Ischemic strokes due to large-vessel occlusions contribute disproportionately to stroke-related dependence and death: a review. Mini review. Front Neurol. (2017) 8:651. doi: 10.3389/fneur.2017.00651, PMID: 29250029 PMC5715197

[11] Wechsler LR Adeoye O Alemseged F Bahr-Hosseini M Deljkich E Favilla C. Most promising approaches to improve stroke outcomes: the stroke treatment academic industry roundtable XII workshop. Stroke. (2023) 54:3202–13. doi: 10.1161/STROKEAHA.123.044279, PMID: 37886850

[12] Smith EE Zerna C Solomon N Matsouaka R Mac Grory B Saver JL. Outcomes after endovascular thrombectomy with or without alteplase in routine clinical practice. JAMA Neurol. (2022) 79:768–76. doi: 10.1001/jamaneurol.2022.1413, PMID: 35696198 PMC9194745

